# Neuro-environmental interactions: a time sensitive matter

**DOI:** 10.3389/fncom.2023.1302010

**Published:** 2024-01-08

**Authors:** Azzurra Invernizzi, Stefano Renzetti, Elza Rechtman, Claudia Ambrosi, Lorella Mascaro, Daniele Corbo, Roberto Gasparotti, Cheuk Y. Tang, Donald R. Smith, Roberto G. Lucchini, Robert O. Wright, Donatella Placidi, Megan K. Horton, Paul Curtin

**Affiliations:** ^1^Department of Environmental Medicine and Public Health, Icahn School of Medicine at Mount Sinai, New York, NY, United States; ^2^Department of Medical and Surgical Specialties, Radiological Sciences and Public Health, University of Brescia, Brescia, Italy; ^3^Department of Neuroscience, Neuroradiology Unit, ASST Cremona, Cremona, Italy; ^4^ASST Spedali Civili Hospital, Brescia, Italy; ^5^Department of Medical Surgical Specialties, Radiological Sciences and Public Health, University of Brescia, Brescia, Italy; ^6^Department of Microbiology and Environmental Toxicology, University of California Santa Cruz, Santa Cruz, CA, United States; ^7^Department of Environmental Health Sciences, Robert Stempel School of Public Health, Florida International University, Miami, FL, United States; ^8^LinusBio, Inc., New York, NY, United States

**Keywords:** resting state, fMRI, machine learning, XGB classifier, exposome analysis

## Abstract

**Introduction:**

The assessment of resting state (rs) neurophysiological dynamics relies on the control of sensory, perceptual, and behavioral environments to minimize variability and rule-out confounding sources of activation during testing conditions. Here, we investigated how temporally-distal environmental inputs, specifically metal exposures experienced up to several months prior to scanning, affect functional dynamics measured using rs functional magnetic resonance imaging (rs-fMRI).

**Methods:**

We implemented an interpretable XGBoost-shapley additive explanation (SHAP) model that integrated information from multiple exposure biomarkers to predict rs dynamics in typically developing adolescents. In 124 participants (53% females, ages, 13–25 years) enrolled in the public health impact of metals exposure (PHIME) study, we measured concentrations of six metals (manganese, lead, chromium, copper, nickel, and zinc) in biological matrices (saliva, hair, fingernails, toenails, blood, and urine) and acquired rs-fMRI scans. Using graph theory metrics, we computed global efficiency (GE) in 111 brain areas (Harvard Oxford atlas). We used a predictive model based on ensemble gradient boosting to predict GE from metal biomarkers, adjusting for age and biological sex.

**Results:**

Model performance was evaluated by comparing predicted versus measured GE. SHAP scores were used to evaluate feature importance. Measured versus predicted rs dynamics from our model utilizing chemical exposures as inputs were significantly correlated (*p* < 0.001, *r* = 0.36). Lead, chromium, and copper contributed most to the prediction of GE metrics.

**Discussion:**

Our results indicate that a significant component of rs dynamics, comprising approximately 13% of observed variability in GE, is driven by recent metal exposures. These findings emphasize the need to estimate and control for the influence of past and current chemical exposures in the assessment and analysis of rs functional connectivity.

## Introduction

Intrinsic functional connectivity of the brain has been widely investigated through the analysis of spontaneous (task- independent) blood-oxygen level dependent (BOLD) fluctuations at rest. Resting-state fMRI (rs) allowed the discovery of multiple functional networks underlying cognitive, behavioral and perceptual processing (Greicius Krasnow et al., 2003; Park and Friston, 2013) and facilitated further understanding of the temporal and spatial correlation patterns of interconnected brain regions. These correlation patterns are observed in controlled conditions to ensure that variability in sensory, perceptual, or behavioral-evoked neural processing is minimal, thus providing a baseline to characterize connectivity patterns at rest. However, this use of task-free data inherently presumes that the impact of the environment on rs signal is essentially concurrent, or minimally lagged, during data acquisition. Here, we investigated how temporally-distal environmental inputs, specifically metal exposures experienced up to several months prior to testing, affect rs functional dynamics.

To fully characterize the impact of metals exposure on the brain, recent studies have begun investigating the combined or synergistic effect of multiple co-exposures, which may better capture the complex exposure landscape encountered in “real-world” circumstance (de Water et al., 2019; Thomason et al., 2019; Bauer et al., 2020). A challenge in this approach, and in any assessment of exposure, is that differential toxicokinetics involved in varying metal exposures yield a heterogenous distribution of metal biomarkers among different biological media. Therefore, each medium (i.e., blood, urine, etc) provides complementary information on different biological processes. To accommodate this reality, recent studies leverage mixtures-based methods to combine information from multiple exposure matrices (Bobb et al., 2015; Levin-Schwartz et al., 2020; Bauer et al., 2021). These metals mixture-based models demonstrated a more-negative impact on neurodevelopment than single metal model (Claus Henn et al., 2012; Claus et al., 2014; Freire et al., 2018; Horton et al., 2018; Invernizzi et al., 2023). A previous study in our group used an integrated measure of metal mixtures across multiple media, called multi-media biomarkes (MMB) (Levin-Schwartz et al., 2020), to analyze the impact on the brain of different neurotoxic metals across multiple media (Invernizzi et al., 2023). Robust associations were found between metals and graph metrics (GE and LE), however the small sample size of the used dataset resulted in relatively small effect sizes and assumption of linear associations between outcomes were made. These results highlight the need for alternative approaches that allow investigating non-linear associations between outcomes and possible multiplicative effects, and do not require a large sample size to allow appropriate cross-validation procedures, limiting their utility in smaller studies.

In this study, we introduce a novel, alternative and interpretable approach to link high-dimensional environmental exposure assessment to functional connectivity utilizing a machine learning (ML) based predictive framework that allows us to: (a) evaluate the model performance based on the predictive efficacy of the model and (b) leverage all available data simultaneously, much as a mixture approach aims to achieve, while implementing a robust leave-one-out cross validation paradigm to ensure generalizability. The XGBoost model used here has the ability to: (i) integrate broad array of hyperparameters useful in preventing overfitting and generating generalizable results, particularly the alpha (L1-type penalization) and subsample hyperparameters used; (ii) forest-based methods are adept at handling and capturing non-linear relationship between variables; and, (iii) xgboost implementation of fast gradient boosting has integrated support for feature importance scores, as is readily-adaptable to SHAP-based methods that allow us to gain more insights into which variable have the most impact on our final predictions (Shwartz-Ziv and Armon, 2021).

To help interpretability, a game-theory based measure of variable importance, the Shapley Additive Explanation (SHAP) (Mangalathu et al., 2020) method was used to evaluate the importance of each feature in our final model. SHAP scores previously have been applied to explore gene–gene and gene–environment interactions. Here for the first time, we applied SHAP scores to investigate brain-environment dependencies.

We leveraged this platform to explore the importance of temporally-distal environmental chemical inputs on rs intrinsic functional connectivity. Utilizing multi-modal exposure assessment combined with a ML-based modeling platform, we generated a predictive model to determine the extent to which contemporaneous rs functional connectivity could be predicted from environmental inputs experienced up to months prior. Critically, our results suggest that the role of past chemical exposures is a critical variable for future rs studies to control for in the evaluation of rs functional connectivity. These findings highlight the utility of leveraging interpretable machine learning algorithms in neuroexposomic investigations for discovering overlooked interactions between the brain and environmental exposures.

## Materials and methods

### Participants

The Public Health Impact of Metal Exposure (PHIME) cohort investigates associations between metal exposure from anthropogenic emissions and developmental health outcomes in adolescents and young adults living proximate to the ferro-manganese industry in northern Italy. Details of the study have been described elsewhere (Lucchini et al., 2012a; Lucas et al., 2015). Inclusion criteria were: birth in the areas of interest; family residence in Brescia for at least two generations; residence in the study areas since birth. Exclusion criteria were: having a severe neurological, hepatic, metabolic, endocrine or psychiatric disorder; using medications (in particular with neuro-psychological side effects); having clinically diagnosed motor deficits or cognitive impairment and having visual deficits that are not adequately corrected. Detailed description of this recruitment process and study design can be found in previous publications (Lucchini et al., 2012a,b). A convenience-based sample of 202 participants (53% female, ages 13–25 years) were selected and willing to participate in a multimodal magnetic resonance imaging (MRI) study, PHIME-MRI. They completed multimodal MRI scans, neuropsychological tests, including measures of IQ (Kaufman Brief Intelligence Test, Second Edition (KBIT-2)) (Kaufman and Kaufman, 2014; Reynolds et al., 2014, 2018), memory and motor functions. All participants satisfied eligibility criteria for MRI scanning (i.e., no metal implants or shrapnel, claustrophobia, no prior history of traumatic brain injury, body mass index (BMI) ≤ 40). Manganese, lead, chromium, copper, nickel and zinc (Mn, Pb, Cr, Cu, Ni, Zn, respectively) were measured in saliva, hair, fingernails, toenails, blood and urine, for each PHIME-MRI participant. Complete exposure data (i.e., all metals in all media for a total of 6 components), MRI and covariates data were available for 124 participants (57% females, with an average age of 19.04 years, range = 13–25) included in this analysis (69 missing biomarkers and 9 poor MRI quality).

Written informed consent was obtained from parents, while participants provided written assent. Study procedures were performed in accordance with relevant guidelines and regulations, approved by the Institutional Review Board of the University of California, Santa Cruz, the ethical committees of the University of Brescia, and the Icahn School of Medicine at Mount Sinai.

### Data availability

De-identified data will be made available upon reasonable request to the corresponding author; raw image files can be accessed upon completion of a data use agreement.

### Biomarker measures of exposure

Biological samples including venous whole blood, spot urine, saliva, hair, fingernails and toenails were collected from each subject upon enrollment, as described in detail in previous studies (Smith et al., 2007; Eastman et al., 2013; Lucas et al., 2015; Butler et al., 2019). Biological samples were processed and analyzed for metal concentrations using magnetic sector inductively coupled plasma mass spectroscopy (Thermo Element XR ICP-MS), as described elsewhere (Smith et al., 2007; Eastman et al., 2013; Lucas et al., 2015; Butler et al., 2019). A complete overview of biomarkers can be found in Table 1, while Pearson’s correlations between biomarkers is reported in Supplementary Figure S1. Samples with values less than the LOD were substituted with LOD/square root of 2 (Ganser and Hewett, 2010).

**Table 1:** Metal concentrations (Mn, Pb, Cr, Cu, Ni, and Zn) measured in blood, urine, hair, saliva, fingernails, and toenails collected from 124 adolescent participants included in the PHIME-MRI study.

Medium*	Metal	GM	GSD	% > LOD	LOD mean ± SE
Saliva	Lead	0.181	3.386	89	0.052 ± 0.004
	Chromium	0.393	3.729	90	0.125 ± 0.005
	Manganese	3.178	3.084	100	0.084 ± 0.001
	Nickel	1.204	3.014	93	0.198 ± 0.011
	Cupper	8.653	2.403	99	0.418 ± 0.034
	Zinc	45.865	2.898	100	2.3189 ± 0.136
Hair	Lead	0.108	2.744	100	0.003 ± 0.001
	Chromium	0.044	2.542	100	0.004 ± 0.001
	Manganese	0.072	2.557	100	0.006 ± 0.001
	Nickel	0.071	4.024	88	0.014 ± 0.001
	Cupper	10.190	1.629	100	0.048 ± 0.003
	Zinc	85.750	1.619	100	0.209 ± 0.008
Fingernails	Lead	0.030	3.326	96	0.003 ± 0.001
	Chromium	0.082	2.348	99	0.004 ± 0.001
	Manganese	0.097	2.244	99	0.006 ± 0.001
	Nickel	0.192	5.522	94	0.017 ± 0.001
	Cupper	2.813	1.523	100	0.046 ± 0.002
	Zinc	39.818	1.702	100	0.209 ± 0.009
Toenails	Lead	0.058	2.530	100	0.003 ± 0.001
	Chromium	0.113	3.113	100	0.004 ± 0.001
	Manganese	0.096	3.081	99	0.006 ± 0.001
	Nickel	0.073	3.756	80	0.017 ± 0.001
	Cupper	2.475	1.352	100	0.046 ± 0.003
	Zinc	26.665	1.763	100	0.208 ± 0.009
Urine	Lead	0.338	1.980	100	0.063 ± 0.003
	Chromium	0.339	2.702	97	0.101 ± 0.004
	Manganese	0.259	3.043	85	0.115 ± 0.003
	Nickel	1.193	2.566	97	0.231 ± 0.007
	Cupper	5.604	1.800	100	0.325 ± 0.012
	Zinc	368.526	1.942	100	4.241 ± 0.302
Blood	Lead	8.409	1.509	100	0.161 ± 0.004
	Chromium	0.442	4.287	75	0.205 ± 0.011
	Manganese	8.364	1.509	100	0.482 ± 0.023
	Nickel	1.901	0.136	81	0.791 ± 0.035
	Cupper	574.459	1.310	100	1.108 ± 0.037
	Zinc	3599.253	1.448	100	7.651 ± 0.636

GM, Geometric mean; GSD, Geometric standard deviation; LOD, limit of detection; SE, standard error of the mean. *Metrics used to measure metal concentrations within each medium were: blood and saliva (ng/mL), hair (ug/g), urine (ug/mL).

### MRI and fMRI data acquisition

Magnetic resonance imaging (MRI) and functional MRI (fMRI) data acquisition was performed on a high-resolution 3-Tesla SIEMENS Skyra scanner using a 64-channel phased array head and neck coil, at the Neuroimaging Division of ASST Spedali Civili Hospital of Brescia. For each participant, a high-resolution 3D T1-weighted structural scan was acquired using a MPRAGE sequence (TR =2,400 ms, TE = 2.06 ms, TI = 230 ms, acquisition matrix = 256×256 and 224 sagittal slices with final voxel size = 0.9 mm^3^). Fifty contiguous oblique-axial sections were used to cover the whole brain where the first four images were discarded to allow the magnetization to reach equilibrium. For each subject, a single 10-min continuous functional sequence using a T2*weighted echo-planar imaging (EPI) sequence (TR = 1,000 ms, TE = 27 ms, 70 axial slices, 2.1 mm thickness, matrix size 108×108, covering the brain from vertex to cerebellum) was acquired. During resting-state scans, lights of the MRI room were off, and participants were instructed to stay awake, relax and daydream (not think about anything) while keeping eyes open. They were presented with an image of a night skyline figure projected on a MRI compatible monitor. Padding was used for comfort and reduction of head motion. Earplugs were used to reduce noise. Data were read by a board-certified radiologist to determine quality and possible incidental findings - no findings were reported.

### fMRI data analyses

Image pre-processing, global efficiency calculation, and statistical analyses were performed using SPM12 (Wellcome Department of Imaging Neuroscience, London, UK), Brain Connectivity toolbox (Rubinov et al., 2009; Rubinov and Sporns, 2010) and customized scripts, implemented in MatLab 2016b (The Mathworks Inc., Natick, Massachusetts) and R (v3.4).

#### Image preprocessing

For each subject, the structural magnetic resonance image was co-registered and normalized against the Montreal Neurological Institute (MNI) template and segmented to obtain white matter (WM), gray matter (GM) and cerebrospinal fluid (CSF) probability maps in the MNI space. FMRI data were spatially realigned, co-registered to the MNI-152 EPI template and subsequently normalized utilizing the segmentation option for EPI images in SPM12. All normalized data were denoised using ICA-AROMA (Pruim et al., 2015). Additionally, spatial smoothing was applied (8 millimeters) to the fMRI data. No global signal regression was applied. Based on the Harvard-Oxford (Desikan et al., 2006) atlas, 111 regions of interest (ROI; 48 left and 48 right cortical areas; 7 left and 7 right subcortical regions and 1 brainstem) were defined. In this atlas, the brain areas were defined using T1-weighted images of 21 healthy male and 16 healthy female subjects (ages 18–50). The T1-weighted images were segmented and affine-registered to MNI152 space using FLIRT (FSL), and the transformations then applied to the individual brain areas’ label. For each ROI, a time-series was extracted by averaging across voxels per time point. To facilitate statistical inference, data were “pre-whitened” by removing the estimated autocorrelation structure in a two-step generalized linear model (GLM) procedure (Monti, 2011; Bright and Murphy, 2015). In the first step, the raw data were filtered against the 6 motion parameters (3 translations and 3 rotations). Using the resulting residuals, the autocorrelation structures present in the data were estimated using an Auto-Regressive model of order 1 [AR (Greicius Krasnow et al., 2003)] and then removed from the raw data. Next, the realignment parameters, white matter (WM) and cerebrospinal fluid (CSF) signals were removed as confounders on the whitened data.

#### Network analysis

Global Efficiency (GE) was computed using the Brain Connectivity toolbox (Rubinov et al., 2009; Rubinov and Sporns, 2010) on the defined ROI time course data per subject. GE builds on the concept of efficient integration of communication in a network at a global level. GE is defined as the inverse of the average characteristic path length between all nodes in the networks (Latora and Marchiori, 2001; Bullmore and Sporns, 2012). For each individual node, with each node defined as an ROI, the shortest number of intermediary nodes required to traverse a path from one node to another was computed. Then, the average number of shortest steps to all defined nodes was computed separately for each node. To correct for the total number of connections between nodes, the inverse of the average number of shortest steps for each node was summed across all network nodes and normalized. GE was scaled from 0 to 1, with a value of 1 indicating maximum GE in observed distribution and then adjusted for age and biological sex (values from −2 to 4).

### Statistical analysis

#### Descriptive statistics

Descriptive statistics (geometric mean, geometric standard deviation, and Spearman’s correlation) were used to characterize the metal concentrations in different media.

#### Predictive modeling

The goal of the predictive modeling was to utilize descriptive statistics (“features”) generated in the descriptive analysis of exposure biomarkers to predict GE metric. The model utilized for predictive classification was a form of ensemble gradient boosting (Chen and Guestrin, 2016), referred to as XGBoost (“Extreme Gradient Boosting”). This approach was selected for the utility of tree-based models for capturing non-linear dependencies and interactions among features, while also leveraging the efficacy of gradient boosting. For hyperparameter tuning, 500 iterations of leave-one-out cross validation were implemented to evaluate the best-performing set of hyperparameters. Following this, the optimal hyperparameter set was used to train a model with leave-one-out cross validation, and the performance of this model evaluated by computing the correlation between predicted GE metric versus measured GE metric in each hold out subject. Based on the Pearson correlation coefficient (r) obtained between the predicted and actual GE value, we finally calculated the explained variance of the model (VE) using the following formula: VE = r^2^ (Park and Friston, 2013).

#### Features importance analysis

Given the ensemble decision trees used in the XGBoost classification algorithm, one feature can be used in multiple locations across the decision trees algorithm making it challenging to interpret the feature importance of the model. For this reason, the Shapley Additive Explanation (SHAP) (Mangalathu et al., 2020) method was used to evaluate each feature’s importance in our final trained model and generate feature importance scores and individual-level explanations for predictions. Based on the trained model, a unique SHAP score was estimated to quantify the contribution of each measurement to model predictions. This score quantifies the effect of each feature on the classification model by measuring the deviation from the average prediction brought by the value of a specific feature. This approach allows the evaluation of non-linear aspects of feature importance; for example, if a given feature contributes to predictive efficacy primarily in cases of extreme scores. In contrast, we subsequently computed the average absolute SHAP value for each feature to capture global importance. To enhance understanding, SHAP values were visualized using bar and beeswarm plots.

#### Software implementation

All descriptive statistical analyses and predictive modeling were implemented using R (version 4.2.1) programming language. The following libraries were used: “data.table” and “imputeTS” for data manipulation; “mlr” and “xgboost” for model training, fitting, and prediction; “SHAPforxgboost” and “ggplot2” libraries were used to quantify and visualize model prediction by computing SHAP scores for each feature, respectively.

## Results

**Figure 1:**
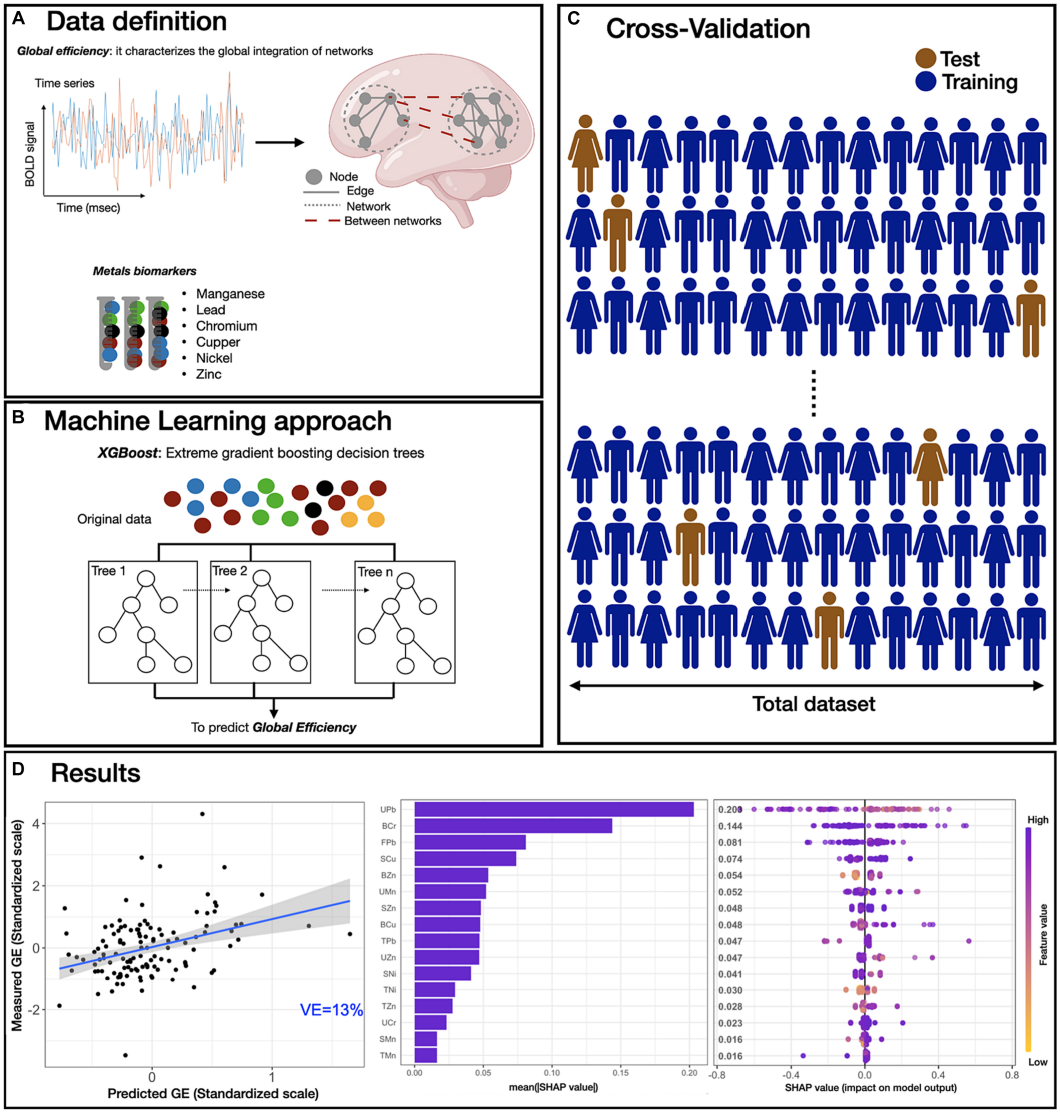
Overview of ML predictive framework. **(A)** Resting-state fMRI data were processed, and the averaged time-series were extracted using the Harvard-Oxford atlas. Then, the global efficiency (GE) metric was computed for each participant. Small solid gray circles represent nodes of the graphs (brain regions), while gray connecting lines are the edges of the graph (functional connections). Larger dotted circles represent segregated sub-graphs/networks at the whole brain level. For the exposure, six biological samples (blood, saliva, hair, urine, fingernails, and toenails) were collected and processed for six metal concentrations (manganese, lead, chromium, copper, nickel and zinc). **(B)** XBoost model was used to predict the GE metric using all exposure biomarkers data (“features”). **(C)** For model training, 500 iterations of leave-one-out cross validation were used, and all features were utilized in the model training. **(D)** The performance of the XGBoost model was evaluated by computing the correlation between predicted GE metric versus scaled GE metric (adjusted for age and biological sex) in the hold out subjects obtaining a *p* < 0.001, *r* = 0.36 and an explained variance (VE) of 13% (Panel a). Then, SHAP scores were computed for all features (metals exposures) used in the model and the average absolute SHAP value was used to quantify the feature importance. In Panel b, each bar shows the mean absolute SHAP value of each feature, sorted in decreasing order. The most impactful features are displayed and higher SHAP score indicates a more significant contribution in the model prediction, in this case in the prediction of GE metric. Panel c shows the beeswarm plot of SHAP values distribution for the highest ranking features of our model. Feature values associated with a single GE prediction are color-coded, yellow/purple corresponding to low/high metals exposure values, respectively. On the x-axis, the SHAP values are shown representing the impact of a feature with respect to the prediction of the GE metric. Features are sorted using the mean absolute SHAP value in descending order with most important features at the top. Each dot corresponds to one subject. Plots are based on the XBoost model with all features included and leave-one-out-cross validation. BOLD: blood oxygen-level dependent. Features abbreviations: the first letter represents the medium (S, saliva; B, blood; U, urine; H, hair; F, fingernails; T, toenails) and the second and third letters represent the metals (Mn, manganese; Pb, lead; Cr, chromium; Cu, copper; Ni, nickel; Zn, zinc).

The complete pipeline of rs fMRI data analysis, model implementation and feature analysis is presented in Figure 1; additional details of the ML model are provided in Material and Methods section. Observed GE was significantly correlated with predicted GE from the XGBoost model trained with all metal concentrations (Mn, Pb, Cr, Cu, Ni, and Zn) measured in blood, urine, hair, saliva, fingernails and toenails as inputs (*p* < 0.001) with a correlation coefficient of 0.36. Based on this correlation, the explained variance of the final model was computed (VE = 13%). To interpret the influence of each feature in the model (i.e., individual metal exposure in each medium) on predicting GE, SHAP scores were calculated to measure feature importance, both at the level of the absolute mean SHAP score (Figure 1B), and in consideration of SHAP scores relative to feature distributions (Figure 1C). These results indicate that urinary and nail Pb, blood Cr, and salivary Cu exposures contributed most to the prediction of global efficiency. Further, analysis of SHAP scores relative to the distribution of urinary Pb (UPb) values (Figure 1C) indicates a negative association between UPb and predicted GE; that is, as UPb increased, the predicted value of GE decreased. In contrast, high blood zinc (BZn) values result in positive SHAP scores, indicating a positive association between BZn and predicted GE.

## Discussion

We present a novel ML-based framework to evaluate the brain’s distinctive response to temporally-distal environmental inputs, specifically metal exposures occurring up to several months prior to scan. Our findings emphasize that traditional perspectives on environmental control, i.e., homogenization of stimulus and behavioral conditions, fail to account for a critical source of environmentally-driven variance among participants. Leveraging the predictive information provided by the ML model together with SHAP scores, we successfully disentangle, interpret and quantify the strong influence of concurrent and recent past metal exposures that explain 13% of current brain dynamics in adolescents. Finally, this method accurately predicts rs metrics and highlights the power of simultaneously using exposomics data and interpretable ML algorithms for discovering overlooked interactions between environmental exposures and the brain.

Environmental neuroscientists typically assess brain-environmental interactions by investigating the association between individual components of exposure, in our case environmental metals, and brain metrics (Berman et al., 2019a,b). Based on their unique chemical properties and similar neurobiological mechanisms of actions, several studies report synergistic neurotoxic effects of metals-exposure (Lopes de Andrade et al., 2021). Metals within our mixture have been shown to produce such synergistic neurotoxic effects (Tao et al., 1999; Chen et al., 2016; Lu et al., 2018), and epidemiological studies suggest that co-exposure to multiple metals, compared to individuals, increases disruption to human neurodevelopment (Kim et al., 2009; Claus Henn et al., 2012; Claus et al., 2014; Levin-Schwartz et al., 2021). Few neuroimaging studies account for this synergistic action of multiple metals mixture on neurophysiological dynamics (Invernizzi et al., 2023) and rather focus on single-metal assessments (de Water et al., 2018, 2019). Here, we applied a multi-modal exposure assessment (i.e., multiple metals in several media) combined with ML-based modeling to investigate the impact of mixed metal exposures on brain dynamics while retaining the discrete information regarding each individual metal.

Our findings reveal a clear link between temporally-distal environmental exposures and current neurophysiological dynamics. When acquiring rs dynamics to assess brain activation patterns, we typically control for sensory and perceptual environments during testing conditions by removing all external stimuli (room lights or visual stimuli) and encouraging the participant to remain still and relaxed and either keep eyes closed or focused on a non-stimulation fixation point (i.e., seascape or cross). Notably, these co-occurring sensory and perceptual inputs operate on the brain signals within timescales of millisecond-to-second intervals as shown in task-based fMRI data (Katwal et al., 2013). In contrast, here we show that chemical environmental inputs, in particular exposures that have occurred weeks and months previously, are also important determinants of neurophysiological dynamics. These results may be particularly relevant in the context of the current reproducibility crisis; that is, neuroimaging studies suffer from high variability and lack of reproducibility (Marek et al., 2022). This challenge may be explained, at least partially, by addressing the impact of overlooked external stressors (i.e., sociodemographic metrics, −omics data, environmental factors) on the functional brain signals. In this study, we confirm that the past chemical environment is certainly critical to control or account for. Accordingly, future studies should consider how environmental, social, and other past exposures might play a role in shaping the recorded brain signals.

Given the high dimensionality of multiple exposures, compounded by multiple exposure media, other typical approaches might be considered (i.e., mixtures-based). More classical mixtures-based approaches [i.e., BKMR (Bobb et al., 2015), WQS (Carrico et al., 2015; Tanner et al., 2019; Curtin et al., 2021)] assess multiple mixtures simultaneously and are applied in an explanatory setting, i.e., either to test hypotheses, or to estimate the effects of contributing chemicals. These methods require a large sample to allow appropriate cross-validation procedures and have not been leveraged in a predictive framework to assess the extent to which past metals co-exposures drive the contemporaneous underlying brain dynamics. An additional strength of our approach is the combination of XGBoost and SHAP scores allowing us to explain the contribution of each feature at both a global level, and at a fine-scale relative to the distribution of each measurement. For example, the distribution of SHAP scores for urinary Pb (UPb), the top contributor to rs dynamics prediction, relative to the observed values of UPb indicated a non-linear association, with high UPb values contributing disproportionally to model efficacy. Globally, UPb values were negatively associated with global efficiency, consistent with previous studies showing high lead levels disrupt neuronal activity (Invernizzi et al., 2023) and are associated with altered structural connectivity and functional activation patterns in children and adults (Bressler and Goldstein, 1991). On the contrary, the SHAP scores for blood Zinc (BZn) revealed a positive linear association in predicting rs dynamics. Zn is a neuroactive metal that can be both neuro-protective and neuro-toxic based on timing, dose, and outcome of interest (Sandstead et al., 2000; Takeuchi et al., 2020; Li et al., 2022). In this case, high BZn values are positively contributing to model efficacy on predicting GE.

Despite the accuracy and interpretation of the ML-based framework presented here, there are several limitations. Our results show a significant correlation between the observed and predicted rs signal from the XGBoost model that trained with all available metal concentrations. However, in this study, we considered only exposure to six metals. Future studies should also consider examining the effect of additional exposures and omics data (i.e., epigenomics, proteomics, transcriptomics and metabolomics) and sociodemographic metrics to capture the social and physical environment and their impact on the functional brain signals. Given the small sample size of our dataset and the limited exposure assessment, it was not possible to validate with independent and external cohorts to further generalize these results. To account for this, we implemented a leave-one-out cross validation (LOOCV) in our model. However, the LOOCV might introduce high variance in the results, as it trains and validates the model on each individual data point. However, this method inherently relies exclusively on in-sample measures of performance, we cannot estimate out-of-sample performance which could capture the generalizability of these results. Future studies should investigate alternative validation techniques such as k-fold cross-validation or bootstrapping that can mitigate some of the variance of LOOCV. Finally, this study focuses exclusively on resting state dynamics, these brain-environment interactions may influence task-based functional signals as well.

Utilizing multi-modal exposure assessment combined with a ML-based modeling, we were able to quantify the impact of the temporally-distal environmental on current neurophysiological dynamics. Our work highlights how this continuous brain-environment interaction is key to advance our understanding of neural mechanisms and can inform on both disease pathogenesis and future public health policies. The impact of overlooked external stressors (i.e., sociodemographic metrics, −omics data, environmental factors) may partially explain the high variability and lack of reproducibility causing the reproducibility crisis in neuroimaging studies, and, when appropriate, future studies should consider how environmental, social, and other past exposures might play a role in shaping the recorded brain signals.

## Data availability statement

The data analyzed in this study is subject to the following licenses/restrictions: De-identified data will be made available upon reasonable request to the corresponding author; raw image files can be accessed upon completion of a data use agreement. Requests to access these datasets should be directed to AI, azzurra.invernizzi@mssm.edu.

## Ethics statement

The studies involving humans were approved by Written informed consent was obtained from parents, while participants provided written assent. Study procedures were performed in accordance with relevant guidelines and regulations, approved by the Institutional Review Board of the University of California, Santa Cruz, the ethical committees of the University of Brescia, and the Icahn School of Medicine at Mount Sinai. The studies were conducted in accordance with the local legislation and institutional requirements. Written informed consent for participation in this study was provided by the participants’ legal guardians/next of kin.

## Author contributions

AI: Conceptualization, Data curation, Formal analysis, Validation, Visualization, Writing – original draft, Writing – review & editing. SR: Formal analysis, Methodology, Writing – review & editing. ER: Methodology, Supervision, Validation, Writing – review & editing. CA: Data curation, Writing – review & editing. LM: Data curation, Writing – review & editing. DC: Data curation, Writing – review & editing. RG: Data curation, Writing – review & editing. CT: Conceptualization, Data curation, Writing – review & editing. DS: Data curation, Writing – review & editing. RL: Data curation, Writing – review & editing. RW: Funding acquisition, Writing – review & editing. DP: Data curation, Funding acquisition, Writing – review & editing. MH: Conceptualization, Funding acquisition, Resources, Supervision, Writing – review & editing. PC: Conceptualization, Methodology, Software, Supervision, Validation, Writing – review & editing.
