# Supplementary material for: Neuro-environmental interactions: a time sensitive matter
Source: Front Comput Neurosci. 2024 Jan 8;17:1302010. doi: 10.3389/fncom.2023.1302010 (PMC10800942; doi:10.3389/fncom.2023.1302010)
Supplement: Supplementary file 1 [file Data_Sheet_1.docx]

**Supplementary Materials**


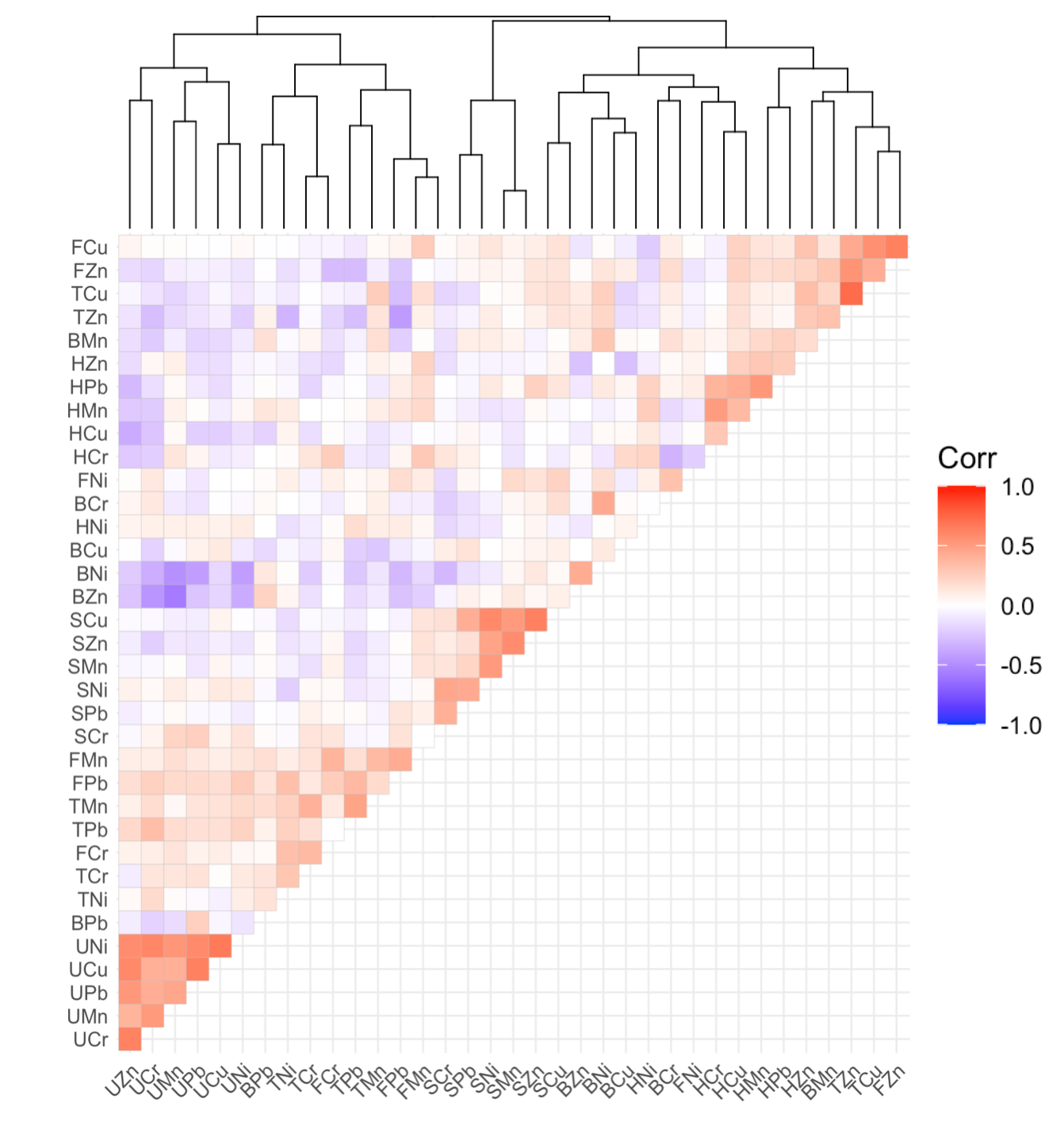


**Figure S1 - Heatmap of metals exposure.**  Spearman's correlations and hierarchical clustering between all biomarkers collected in PHIME-MRI**.** Components abbreviations: the first letter represents the medium (S=saliva, B=blood, U=urine, H=hair, F=fingernails, T=toenails) and the second and third letters represent the metals (Mn=manganese, Pb=lead, Cr=chromium, Cu=copper, Ni=nickel, Zn=zinc).
